# Toxicity, Phytochemical Composition, and Enzyme Inhibitory Activities of Some Indigenous Weed Plant Extracts in Fruit Fly,* Drosophila melanogaster*

**DOI:** 10.1155/2018/2325659

**Published:** 2018-04-12

**Authors:** Bushra Riaz, Muhammad Kashif Zahoor, Muhammad Asif Zahoor, Humara Naz Majeed, Irum Javed, Aftab Ahmad, Farhat Jabeen, Muhammad Zulhussnain, Kishwar Sultana

**Affiliations:** ^1^Department of Zoology, Government College University Faisalabad, Faisalabad, Pakistan; ^2^Department of Microbiology, Government College University Faisalabad, Faisalabad, Pakistan; ^3^Department of Biochemistry, Government College Women University Faisalabad, Faisalabad, Pakistan; ^4^Centre of Department of Biochemistry/US-Pakistan Center for Advance Studies in Agriculture and Food Security (USPCAS-AFS), University of Agriculture Faisalabad, Faisalabad, Pakistan

## Abstract

*Drosophila melanogaster *being used as model organism is considered as pest of homes, restaurants, and fruit markets. The damaged fruits are also reported to serve as a carrier for various diseases. The current study was designed to evaluate the toxicity of petroleum extract of some weed plants, namely,* Euphorbia prostrata*,* Parthenium hysterophorus*,* Fumaria indica*,* Chenopodium murale*, and* Azadirachta indica, *against* D*.* melanogaster*. Mortality at 10, 20, and 30% concentrations after 24 and 48 hours was found comparatively low.* E. prostrata *caused high mortality (51.64%) at 30% concentration and was found more toxic (LC_50_ 27.76; *P* value 0.00) after 72 hours.* A. indica *showed high LC_50_ value (*P* value 0.15) compared to other weed plants. The combination of* E. prostrata *and* Bti* showed highest mortality (100%; LC_50_ 12.49; *P* value 0.00) after 72 hours. Similarly, the same combination caused maximum reduction in the activity of AChE, AcP, AkP, *α*-Carboxyl, and *β*-Carboxyl enzymes. Phytochemical analysis showed the presence of flavonoids, saponins, tannins, steroids, cardiac glycosides, alkaloids, anthraquinones, and terpenoids. FTIR analysis of* E. prostrata *showed the presence of phenolic compounds. It is suggested that further studies are needed in order to incorporate weed plant extracts in combination with* Bti* for the management of fruit flies.

## 1. Introduction

Fruit flies are considered as serious pests and the cost of infestation has been estimated to millions of dollars annually worldwide [1, 2]. Many species of fruit flies such as* Bactrocera* and* Drosophila* are reported to attack different fruits particularly mango and guava in Pakistan. This causes a major economic threat because of rejection of consignments of mangoes exported at international level [3].

Infestation due to fruit flies is manifested initially by scars in the fruit surface left by stinging through ovipositor of females. As eggs hatch time is very short approximately one day, larvae in a little while start feeding inside the fruit. Within 2 or 3 days, the fruit begins to collapse around the feeding site. Thereafter, mold and invasion by secondary pests may aid to further damage [3].* D. melanogaster* being a versatile model in various biological studies also acts as a fruit pest in nature. It is also acting as a vector of life-threatening pathogen,* Staphylococcus aureus* [4]. Biological control of fruit flies is mainly focused on predacious and parasitic natural enemies, and it has played an essential role in ecological conservation as well as biological control programs. Besides natural enemies, microorganisms and plant extracts have also been used for better management of flies [5]. In addition, fungi and bacteria are also reported to produce reasonable results [6].

Weeds are being investigated for their phytochemical, pharmacological, and biological properties [3, 7, 8]. Recently, insecticidal properties of weeds have been reported in many insects [9–13].* Euphorbia prostrata* is an annual herb used for fever, for bleeding hemorrhoids, and against various abdominal diseases [14]. It is also used as an antidote for venomous bites of wasps and scorpions.* Chenopodiastrum murale* (L.) is widespread noxious herbaceous weed which is reported to show antioxidant and antibacterial activities [15]. The chemical composition showed that extract from* C. murale* had essential oils, flavonoids, sterols, alkaloids, and coumarins which exhibited antibacterial, antifungal, phytotoxic, and insecticidal activities [7, 8].* Parthenium hysterophorus* weed is widely distributed in America, Asia, Africa, and Australia. Its health benefits include remedy for skin inflammation, rheumatic pain, diarrhoea, urinary infections, dysentery, malaria, and neuralgia. It contains various allelochemicals such as glycoside parthenin, hysterin, ambrosin, flavonoids, sitosterol, and some unidentified alcohols [16, 17]. Subsequently, the use of* P. hysterophorus* as biopesticides has also been reported [16].* Fumaria indica* (Hausskn.) (Fumariaceae) is a small, scandent, branched, annual herb and locally known as “Shahtra.” It is regarded as laxative, diuretic, and diaphoretic. It is beneficial in fever, influenza, dyspepsia, liver disorders, and skin infections and also reported as blood purifier. It is also used in syphilis, scrofula, leprosy, constipation, ague, and jaundice. It contains a number of compounds including seven alkaloids. Importantly, it has been reported as safe during acute and chronic toxicity studies [18].

Toxicity of various insecticides including insecticidal activity of alkaloids has been reported in* D. melanogaster* [12, 19, 20].* A. indica *(Neem) extract has also been reported for its toxicological evaluations in comparison to insecticides in* D. melanogaster* [21]. However, no work has been reported yet in Pakistan to control fruit flies through using weed plant extracts. Thus, taking advantage of* D*.* melanogaster *as both an easy model to rear and a fruit pest as well in order to devise a control program for other fruit flies too, the present study was designed to use* Azadirachta indica*,* Euphorbia prostrata*,* Parthenium hysterophorus*,* Fumaria indica*, and* Chenopodium murale *L. for their toxicological studies in* D*.* melanogaster. Bti* was also used along with the extracts as a potent controlling agent. This study serves as the first research work which is conducted using weed extracts against fruit flies in Pakistan.

## 2. Materials and Methods

Research work was performed in the Entomology Lab. Government College University Faisalabad (GCUF), to investigate the efficacy of the selected weed plants against* D. melanogaster. *The fruit fly,* D. melanogaster, *was collected from GCUF and reared using artificial food (Agar, Yeast, Banana, Maltose, Cornmeal, and Sodium Benzoate) [22, 23].

### 2.1. Preparation of Plant Extracts

Common weeds were collected from the rural area of Faisalabad city. Four weed plants extracts and one* A. indica* (Neem plant) extract was used (Table 1). The whole plants were washed thoroughly with clean water and then shade dried for 7-8 days [11]. Dried plants were again oven dried at 60-degree centigrade for 20 minutes. Plants were powdered using grinder machine into fine powder. For oil extract, 100 g of ground sieved sample and 300 ml of petroleum ether (40–60%) were mixed in 1 : 3 in conical flask. These flasks were fixed on rotary machine at 220 rpm for 24 hours. After this the plant extract was filtered through Whatman No. 1 filter paper. From stock solution, working solutions of 10, 20, and 30% concentrations were prepared for each plant [11, 13].

### 2.2. Mortality Bioassay

Mortality bioassay was conducted under standard conditions (Temp. 25°C and RH 80%) using five plant extracts with three different concentrations. In each trial, 20 third instar larvae of* Drosophila* were introduced containing feed and plant extracts with 10% to 30% concentrations. For control, only feed and petroleum ether with the corresponding concentration of extracts were used. Mortality of* Drosophila* larvae was observed after time interval of 24, 48, and 72 hours. Each concentration was replicated three times. Further* Bacillus thuringiensis israelensis (Bti)* was used to compare its efficacy with plant extract. From the observed data percentage mortality was counted by using the following formula [24].

(1) Percentage  mortality=number  of  dead  larvaenumber  of  larvae  tested×100

### 2.3. Enzyme Assay

#### 2.3.1. Preparation of Whole Body Homogenate

For enzymatic estimation, the larvae of* D. melanogaster *were washed thoroughly with distilled water and the adhering water was removed by using bloating paper. The larvae were homogenized using ice-cold sodium phosphate buffer (20 mM, pH 7.0) with the help of Teflon hand homogenizer. Then, the homogenate was centrifuged at 8000 ×g and 4°C for 20 minutes and supernatant was used for the estimation of Esterases or Phosphatases. Solutions and glassware used for homogenization were kept at 4°C prior to use, and the homogenates were held on ice until used for various assays [25].

### 2.4. Quantitative Determination of Esterases and Phosphatases

*(1) Estimation of Acetylcholinesterase Activity*. In the 50 *μ*l of enzyme solution, 50 *μ*l of acetylcholine chloride (2.6 mM) as a substrate and 1 ml of sodium phosphate buffer (20 mM, pH 7.0) were added. It was incubated at 25°C for 5 mins. Then 400 *μ*l of 0.3% Fast blue B salt was added to stop reaction. Blank and sample were run through spectrophotometer. Optical density (OD) was recorded at 405 nm [25].

*(2) Estimation of Carboxylesterase Activity*. The activity of *α*-carboxylesterase and *β*-carboxylesterase was measured in larvae [26]. In 50 *μ*l enzyme solution (homogenates), 1 ml of sodium phosphate buffer (20 mM, pH 7.0) and 50 *μ*l of each *α*-naphthyl acetate and *β*-naphthyl acetate (substrate) were added separately to determine the activities of *α*-carboxylesterase and *β*-carboxylesterase, respectively. The solutions were incubated at 30°C for 20 mins. After incubation, 400 *μ*l of freshly prepared 0.3% Fast blue B in 3.3% SDS was added in each reaction mixture to stop the enzymatic reaction and the color was allowed to develop for 15 min at 20°C. Blank and sample were run on spectrophotometer. Optical density (OD) was recorded at 430 and 590 nm for *α*-carboxylesterase and *β*-carboxylesterase, respectively [25]. 

*(3) Estimation of Acid and Alkaline Phosphatase Activity*. The level of acid and alkaline phosphatases was measured in larvae [27]. The acid phosphatase activity was estimated by mixing 50 *μ*l larval homogenate with 50 *μ*l sodium phosphate buffer (50 mM, pH 7.0) and 100 *μ*l of 20 mM p-nitrophenyl phosphate (substrate). For the estimation of alkaline phosphatase activity, 50 *μ*l larval homogenate was mixed with 50 *μ*l Tris HCl buffer (50 mM, pH 9.0) and 100 *μ*l of 20 mM p-nitrophenyl phosphate (substrate). After that, both solutions of acid phosphatase and alkaline phosphatase were incubated at 37°C for 15 mins in water bath, and the enzymatic reaction was stopped by adding 0.5 N NaOH solution. The absorbance (OD) of the resulting clear supernatants of sample and blank was recorded at 440 nm [25].

The percentage inhibition of the enzyme activity by the test extracts was calculated as follows:

(2) %  enzyme  inhibition=OD  of  control  larvae−OD  of  treated  larvae OD  of  control  larvae×100

### 2.5. Preliminary Phytochemical Screening of the Plant Extracts

The plant extracts prepared by using the petroleum ether as solvent as mentioned above were concentrated by evaporation and stored at 4°C in air tight containers for further experimental studies. Qualitative phytochemical analysis of plant extracts (petroleum ether) was carried out by using standard procedures [28–30].

### 2.6. Test for Alkaloids

One ml of 1% HCl was added to 3 ml of plant extract in a test tube and was treated with few drops of Meyer's reagent (potassium mercuric iodine solution). Formation of white yellowish turbidity or precipitate indicates the presence of alkaloids [31].

### 2.7. Test for Terpenoids (Salkowski Test)

Five ml plant extract was taken in test tube with 2 ml of chloroform (CHCl_3_), and 3 ml concentrated H_2_SO_4_ was carefully added to form a layer. Reddish brown color at interface indicated the presence of terpenoids [28].

### 2.8. Test for Saponins (Foam Test)

In a test tube, 0.5 ml of plant extract was shaken vigorously with 2 ml of distilled water to obtain a stable persistent froth. Foamy lather, if it persists for 10 minutes, indicates the presence of saponins [30, 32].

### 2.9. Test for Flavonoids

Few drops of sodium hydroxide (NaOH) solution were added to the extract in a test tube. A yellow coloration, which becomes colorless on addition of dilute acid, indicated the presence of flavonoids [32, 33].

### 2.10. Test for Tannins

Half ml of crude extract was taken in a test tube, and 1 ml of distilled water and 1-2 drops of ferric chloride solution were added into it. Appearance of blue and greenish black coloration was an indication of gallic tannins and catecholic tannins, respectively [34]. 

*Gelatin Test*. To the test solution were added 1 ml of 1% gelatin solution and 1 ml of 10% NaCl, and white precipitate of gelatin indicates the presence of tannins [29, 30].

### 2.11. Test for Cardiac Glycosides (Keller-Killani Test)

In a test tube, 5 ml of plant extract, 2 ml of glacial acetic acid, and few drops of ferric chloride solution were added. 2 ml of concentrated H_2_SO_4_ was added along the side of test tube. Formation of a brown ring at the interface indicated the presence of glycosides [29].

### 2.12. Test for Phenols

*Ferric Chloride Test*. 3-4 drops of ferric chloride solution were added to crude extract in test tube and shaken well. Formation of bluish black color indicated the presence of phenol [29].

### 2.13. Test for Quinones

To the 2 ml of test substance conc. H_2_SO_4_ was added and shaken well for 5 min, and appearance of red color indicates the presence of quinone [29].

### 2.14. Test for Steroids Liebermann Test

10 ml of chloroform was added to the test solution and then filtered. In a test tube containing 2 ml filtrate, 2 ml of acetic anhydride and few drops of con. H_2_SO_4_ were added. Formation of blue green ring indicates the presence of steroids [33].

### 2.15. Fourier Transform Infrared Spectroscopy (FTIR) Analysis

The* E. prostrata *extract which showed highly significant results was examined by FTIR spectroscopy for the detection of the specific functional groups. The extracts prepared in n-Hexane were frozen at −80°C followed by lyophilization. Infrared absorption spectrum of the lyophilized extract was recorded on a FTIR spectrophotometer (Alpha, Bruker, California, USA) in the region 4000 to 500 cm^−1^ [35, 36].

### 2.16. Statistical Analysis

After calculating percentage mortality, the data for different concentrations were subjected to Probit Analysis program (version 1.5) to determine the LC_50_. ANOVA was performed using Statistica 13.0 for windows. The means were separated through Tukey's HSD (Honest Significant Difference) test at a significance level of 0.05. A value of *P* < 0.05 was considered statistically significant.

## 3. Results 

### 3.1. Mortality of* Drosophila melanogaster* Larvae

The mortality data of* D. melanogaster* larvae were observed at various concentrations and exposure time for five plants extracts (Table 2).* E. prostrata* showed high mortality (51.64%) at 30% concentration after 72 hours. Mortality at concentration of 10%, 20%, and 30% after 24 and 48 hours is comparatively low compared to mortality after 72 hours. With* C. murale*, 40.80% mortality was observed at 30% after 72 hours of exposure time. Similarly, low mortality was found at concentration of 10, 20, and 30% after 24 and 48 hours compared to mortality after 72 hours of exposure (Table 2).* A. indica* showed 42.45% mortality at 72 hours, which is higher than mortality after 24 and 48 hours at concentration of 10, 20, and 30%, respectively.* F. indica *showed 43.92% mortality at concentration of 30% after 72 hours which is the second highest mortality among all five extracts. Mortality at 10, 20, and 30% concentration after 24 and 48 hours was comparatively less than the mortality after 72 hours.* P. hysterophorus *showed 41.87% mortality at concentration of 30% after 72 hours. It was found that increased concentration of weeds plant extracts caused high mortality (Table 2).

The LC_50_ value for all five extracts was decreased with the passage of time and the lowest LC_50_ value was observed after 72 hours as compared to 24 and 48 hours against* D. melanogaster.* The LC_50_ value after 72 hours for all five extracts is shown in Table 3. Among all five extracts,* E. prostrata *showed lowest LC_50_ value (27.763; *P* value 0.00) at 72 hours followed by* Fumaria indica *(LC_50_ 36.22; *P* value 0.00). Similarly,* C. murale* and* P. hysterophorus *showed LC_50_ = 39.68 (*P* value 0.01) and LC_50_ = 39.73 (*P* value 0.00), respectively, while* A. indica *showed high LC_50_ value (39.50; *P* value 0.15) compared to other weed plants at 72 hours.

*E. prostrata *showed lowest LC_50_ values (Table 3) and, therefore, was further selected for combinatorial trials with* Bti*. The mean mortality of* E. prostrata *in combination with* Bti *is shown in Table 4.* Bti* solution and* E. prostrata *extract with the concentration of 250 ppm showed highest mortality (100%) after 72 hours. After 24 and 48 hours, 79% and 96% mortality were observed, respectively. The mortality at 100 ppm of* Bti* solution and* E. prostrata* extract was observed as 97 and 90% after 72 and 48 hours and 63% after 24 hours. The mortality shown by 50 ppm* Bti* and* E. prostrata *extract was 88 and 84% after 72 and 48 hours, respectively, whereas 39% mortality was found after 24 hours. Low mortality among all concentrations of* Bti* solution and* E. prostrata *extract was shown by 10 ppm of* Bti* and* E. prostrata *extract, that is, 74% after 72 hours, 69% after 48 hours, and 37% after 24 hours (mean mortality %age). Different control trials were also performed in order to establish the mortality to exclude the effect of physical and/or any other factors. The overall results showed high mortality with combination of* E. prostrata *and* Bti *(250 ppm) compared to the control groups (Table 4).

The results of lethal concentration of 50% mortality (LC_50_) using the combination of* E. prostrata *and* Bti* along with control treatments are shown in Table 5. In combination with* Bti, E. prostrata* showed high toxicity with low LC_50_ values found to decrease with time. The LC_50_ value was 12.49 after 72 hours, whereas after 24 and 48 hours LC_50_ values were found as 75.53 and 34.96, respectively, at all exposure times. Moreover, it was found that mortality increased with an increase of* E. prostrata* and* Bti* concentrations (Tables 4 and 5).

The effect of these weed plant extracts on the enzymatic activity in* D. melanogaster* larvae was observed at various concentrations after 72 hours. The maximum decrease was observed at 30% concentration of extracts.* E. prostrata *induced a decrease in acetylcholine esterase, AcP, AkP, *α*-Carboxyl, and *β*-Carboxyl enzymes by 53.7%, 33.3%, 23.6%, 70.3%, and 61.3%, respectively, with 30% concentration. In addition, a decrease in the activity of acetylcholine esterase (53.01%), AcP (31.06%), AkP (20.99), *α*-Carboxyl (64.17%), and *β*-Carboxyl (47.41%) was found with 30% concentration of* A. indica *extract. A maximum decrease in the activity of AChE (60.31%) by* C*.* murale*, AcP (31.06%) by* E. prostrata*, AkP (25.55%) by* F. indica*, and *α*-Carboxyl (70.39) and *β*-Carboxyl (61.3%) by* E. prostrata* was recorded at 30% concentrations, respectively. It was found that* E. prostrata* induced maximum reduction in the enzymatic activity of* D. melanogaster* larvae (Table 6).

Table 7 shows the percent inhibition of the enzymatic activity of* D. melanogaster* larvae by the combination of* E. prostrata *and* Bti* after 72 hours. The concentrations of 250 ppm caused maximum reduction in the activity of Ache, AcP, AkP, *α*-Carboxyl, and *β*-Carboxyl enzymes. It can be observed that enzyme inhibition was increased with increased concentration of* E. prostrata *and* Bti* (Table 7).

### 3.2. Phytochemical Analysis of Plant Extracts

The whole weed plants extracted in petroleum ether as solvent were investigated for their phytochemical components, that is, flavonoids, saponins, tannins, steroids, cardiac glycosides, alkaloids, anthraquinones, and terpenoids. The qualitative tests revealed the presence of flavonoids (Fl), saponins (Sa), and alkaloids (Al) in all tested extracts.* P. hysterophorus* and* F. indica* showed no sign of tannins (Tn), while steroids (St) were absent in* E. prostrata*. Cardiac glycosides (CG) were not detected in the extract of* F. indica*. Anthraquinones (Anth) were found absent in* C. murale*. Terpenoids were not observed in* E. prostrata *and* C. murale *(Table 8).

### 3.3. Fourier Transform Infrared Spectroscopy (FTIR)

**Figure 1:**
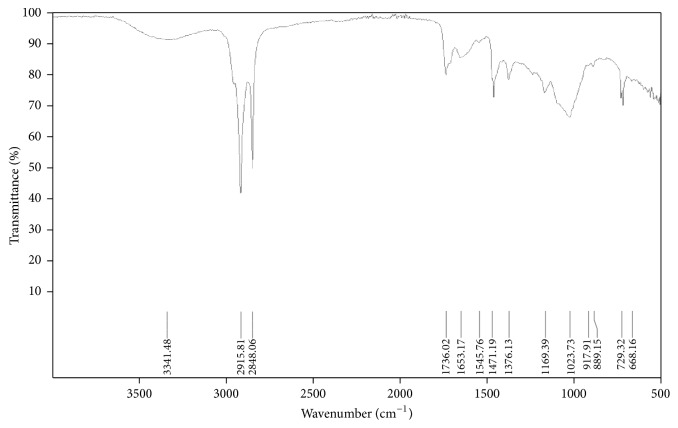
*FTIR spectrum of Euphorbia prostrata*. The spectrum shows a range of 4000 to 500 cm^−1^ wave number (along *x*-axis) and the function of % transmittance (along *y*-axis). Following peaks can be observed: 2915.81 cm^−1^ CH group stretching due to CH_3_, 1736.02 cm^−1^ C=O stretching, 1376.13 cm^−1^ C-N bond vibration, 1169.39 cm^−1^ C-O bond vibration, and 729.32 cm^−1^ C-X bond vibration.

**Figure 2:**
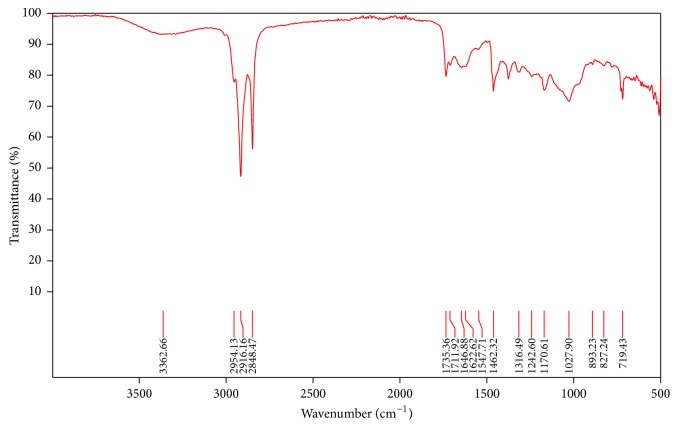
*Full FTIR spectrum of Chenopodium murale*. The spectrum shows a range of 4000 to 400 cm^−1^ wave number (along *x*-axis) and the function of % transmittance (along *y*-axis). Following peaks were observed: 1027.90 cm^−1^ aromatic in-plane C-H bending, 1622.62 cm^−1^ carbonyl (C=O) group, 2916.16 cm^−1^ asymmetric CH_2_ stretching, and 3363.66 cm^−1^ O-H stretching. It reveals the presence of phenolics compounds having carboxylic acids.

Fourier transform infrared spectroscopy (FTIR) is a powerful molecular spectroscopic tool which helps in both quantitative and qualitative analysis of diverse inorganic and organic compounds. It gives results in the form of absorption spectrum. Generally, FTIR looks at the vibration of functional groups present in organic molecules and explores the structural alterations as the function of shifts in wave number [35]. The FTIR spectroscopic analysis of* E. prostrata *extract revealed the presence of various chemical constituents (Figure 1). The absorption at 3341.48 shows the presence of polyphenolic compounds due to presence of flavonoids. The intense absorption bands at 2915.81 cm^−1^ CH group stretching due to CH_3_ and 1736.02 cm^−1^ represent C=O stretching. The absorption band at 1376.13 cm^−1^ denotes the presence of C-N bond vibration possibly due to NH_2_, =NH, or ≡ N absorption. The bands at 1169.39 cm^−1^ show C-O bond vibration (carbonyl group) and 729.32 cm^−1^ C-X bond vibration due to alcoholic or phenolic compounds. The presence of phenolic compounds is also shown in Figure 2 for* Chenopodium murale.*

## 4. Discussion

The chemical insecticides have been considered as the good option for the control of insect pests; however, chemical use causes adverse effects to the biotic and abiotic environment. The choice of new insecticides that meet the requirements of economy, safety, and efficacy highlights the objectivity of farmers. Thus, nowadays biopesticides are being used to control various insect pests [4, 21, 37]. Biopesticides are a type of pesticide derived from natural materials like animals, plants, bacteria, viruses, and certain minerals. The plant kingdom has been recognized as the most efficient producer of chemical compounds, synthesizing many products that are used to defend plants against different pests [38]. Moreover, it has been reported that* Bacillus thuringiensis israelensis *and* pseudomonas* can be successfully used against insect pests including* Drosophila* [6]. Thus, the present study was designed in order to find out a potent weed plant in combination with* Bti *strain to be used for the control of flies as well as a guideline model for other insect pests.

Four different weed plants extracts, namely,* E. prostrata*,* C. murale L*,* F. indica, *and* P. hysterophorus*, were used along with* A. indica* (Neem plant) as a reference to observe their efficacy against* Drosophila melanogaster*. This current study signifies the use of unwanted plants which are considered as pests themselves. It is also worth mentioning that these weed plants act as a rich source of different bioactive compounds that can be biodegraded into nontoxic products which are potentially suitable for integrated pest management [37]. In fact, many researchers reported the effectiveness of plant essential oils and extracts and described the plant based compounds from* A. indica *due to which this plant was taken as a reference for the current research work [9, 39–41].* E. prostrata* showed 51.64% mortality at 30% concentration after 72 hours and showed significant results against* Drosophila *flies.

It has been already reported that methanolic plant extracts caused mortality in fruit fly,* D. melanogaster* [3]. Similarly, the ovicidal and larvicidal activity of essential oils from rosewood and garlic against* Plutella xylostella* was reported [42]. Subsequent studies also showed that* A. indica *caused significant mortality in* D. melanogaster* [21]. However, in the present study,* E. prostrata *and* P. hysterophorus* did not show significant difference of mortality from* A. indica *both at 24 and at 48 hrs (data not shown). Overall* E. prostrata *produced highly significant results compared to other three weed plants. Therefore, it was selected for further trials with* Bti*. Different ppm solutions of* Bti* were used and it was found that increasing the concentration of* Bti *resulted in increased mortality %age. The combination trial of* E. prostrata *plus* Bti *when compared with control trials showed more mortality compared to individual weed plant* E. prostrata* extract and* Bti*. Importantly, 100% mortality was found with* E. prostrata *extract plus* Bti*. LC_50_ values calculated through Probit Analysis showed that combination of* E. prostrata* extract and* Bti *250 ppm has significant results (*P* values 0.00) (Tables 4 and 5).

Plant allelochemicals are useful in increasing the effect of biological control agents [1, 43]. Since these plant chemicals are less expensive, easily biodegradable, and considered as highly suitable for integrated pest management programs being active against a number of insect pests, so they could lead to developing safer insect pest control agents [9–11, 13, 37]. These findings are in agreement with Sultana et al. [9]. They investigated the efficiency of petroleum ether extracts of weed plants,* Euphorbia prostrata *and* Chenopodiastrum murale *for the control of* Trogoderma granarium*. They found relatively high rate of larval mortality after 6 days with 10%, 20%, and 30% extract concentrations. However, at 30% concentration, the corresponding mortality rates induced by* E. prostrata *and* C. murale *are high.

The petroleum ether extract of weed plants also induced enzyme inhibitory activity against* D. melanogaster*. The inhibitory activity of acetylcholinesterase enzyme was reported due to insecticides [44]. In addition, it was also described that acetylcholinesterase is the most sensitive enzyme affected by the insecticides [45]. Furthermore, the inhibition of esterase activity was reported in insects by plant products [46]. Subsequently, a significant reduction in the activity of acetylcholinesterase, total esterase (TE), and arylesterase (AE) was also described in 4th instar larvae of* T. granarium* treated for 80 h exposed period with Phosphine [47].

Similarly, the biochemical effects of seven culinary and medicinal plant oils were described, namely, garlic (*Allium sativum *L.), onion (*Allium cepa *L.), olive (*Olea europaea *L.), rosemary (*Rosmarinus officinalis *L.), sunflower (*Helianthus annuus *L.), peppermint (*Mentha piperita *L.), and camphor (*Eucalyptus globulus*) against* Trogoderma granarium *4th instar larvae [25]. A decrease in glucose and lipid contents and higher protein contents were observed in the treated larvae. It was also found that these plant oils caused less alkaline phosphatase (AKP) activity and low acid phosphatase (ACP) content. Cholinesterase was found to be increased whereas alanine aminotransferase (ALT) and aspartate aminotransferase (AST) activity in 4th instar larvae were found to be decreased. The current results are also in accordance with [48] who reported that* Artemisia annua* extract inhibited the AChE activity in higher doses in treated Sunn pest.

The current findings of IR spectra of* E. prostrata *are in accordance to the previously reported analysis [49]. The phytochemical screening revealed the presence of flavonoids, terpenoids, and tannins. Moreover, IR spectra showed the OH, CH stretching and C=O, C=C, NO_2_, C-N, C-O stretching, respectively. Subsequently, FTIR was used to analyze the various components from different medicinal plants [36]. IR spectra showed the presence of phenolic compounds which were further evaluated for their antioxidant activity. The peak values through FTIR analysis of* Euphorbia prostrata *indicated the presence of phenolic compounds which are consistent with the previous findings [50]. They obtained similar IR spectra corresponding to different functional groups with similar band stretching. Thus, on the basis of absorption values, the presence of polyphenolics (-OH) and flavonoid type compounds was confirmed in the methanolic extract. The plants which showed significant results in the current study contain mainly flavonoids, tannins, and phenolic compounds. It has been reported that these compounds can be potentially used for the control of insect pests of various crops [37].

Although the efficacy of phytochemicals from various plants to evaluate the toxicity has been reported in* D*.* melanogaster*, however, efficacy of* Euphorbia prostrata* and their effect on enzymatic activity in* D*.* melanogaster* have not been reported yet. Thus, the present study is the first report to describe these parameters. In future, further studies can be conducted to extract and characterize the potential phenolic compound found in* E. prostrata *to use in the insect pest management program.

## 5. Conclusion

*Euphorbia prostrata *and* Parthenium hysterophorus *extracts showed toxicity against* Drosophila melanogaster.* However, based on LC_50_ values (*P* values),* E. prostrata *weed plant extract was used along with* Bti *for further trials and highest mortality of* D. melanogaster *was found. It was also found that* E. prostrate* induced maximum reduction in the enzymatic activity of AChE, AcP AkP, *α*-Carboxyl, and *β*-Carboxyl in* D. melanogaster* larvae. Phytochemical analysis showed the presence of flavonoids, saponins, tannins, steroids, cardiac glycosides, alkaloids, anthraquinones, and terpenoids. Moreover, FTIR analysis showed that* E. prostrata *contains phenolic compounds which have been reported to show insecticidal activity.* E. prostrata *plant is easily available in Pakistan and its extract could be greatly used to control fruit flies. In future, further studies are needed to extract and characterize the particular potential phenolic compound found in* E. prostrata *to use in insect pest management programs.

## Figures and Tables

**Table 1:** List of weed plants used during the study.

Number	Scientific name	Common name	Family name	Used parts
1	*Euphorbia prostrata *	Bakkar booti	Euphorbiaceae	Whole plant
2	*Chenopodium murale *L.	Kurand	Amaranthaceae	Whole plant
3	*Azadirachta indica*	Neem	Meliaceae	Whole plant
4	*Fumaria indica*	Shahtra	Papaveraceae	Whole plant
5	*Parthenium hysterophorus*	White top	Asteraceae	Whole plant

**Table 2:** Mean mortality of *Drosophila melanogaster *larvae after 72 hours of exposure to different concentration of weed plants extracts.

Plant name	Concentration		
	10%	20%	30%
*Euphorbia prostrata* (*F* = 13.30; d.f. = 2; *P* < 0.05)	34.06 ± 5.45^a^	42.70 ± 5.28^ab^	51.64 ± 6.25^c^
*Chenopodium murale* (*F* = 13.46; d.f. = 2; *P* < 0.05)	25.38 ± 5.02^a^	33.54 ± 5.44^a^	40.80 ± 6.17^b^
*Azadirachta indica* (*F* = 12.14; d.f. *= *2; *P* < 0.05)	27.49 ± 6.36^a^	34.66 ± 4.99^b^	42.45 ± 5.33^b^
*Fumaria indica* (*F* = 10.77 d.f. = 2; *P* < 0.05)	27.26 ± 5.70^a^	35.97 ± 5.76^b^	43.92 ± 5.56^b^
*Parthenium hysterophorus* (*F* = 9.82; d.f. *=* 2; *P* < 0.05)	26.15 ± 4.19^a^	32.28 ± 5.65^a^	41.87 ± 5.42^b^

a, b, c, and ab: means sharing the same letter within each treatment are not statistically different.

**Table 3:** Toxicity of weed plant extracts against *Drosophila melanogaster *larvae after 72 hours of exposure.

Plants	*N*	LC_50_ (lower value ± upper value)	Slope ± SE	*X* ^2^	*P* value
*Euphorbia prostrata*	20	27.76 (21.47 ± 55.12)	0.0231259 ± 0.0090030	0.0009525	0.00
*Chenopodium murale* L.	20	39.68 (29.66 ± 126.75)	0.0222512 ± 0.0092747	0.0587030	0.01
*Azadirachta indica*	20	39.50 (29.05 ± 180.91)	0.0205005 ± 0.0092029	0.0193744	0.15
*Fumaria indica*	20	36.22 (27.70 ± 94.38)	0.0230412 ± 0.0091880	0.0220428	0.00
*Parthenium hysterophorus*	20	39.73 (29.68 ± 127.85)	0.0221777 ± 0.0092583	0.0793358	0.00

**Table 4:** Mean mortality of *Drosophila melanogaster* larvae exposed to different concentrations of *Bti *and 30% concentrations of *Euphorbia prostrata*.

Number	Concentrations	Exposure time		
		24 h	48 h	72 h
1	Water* + *food	4.44 ± 1.76^a^	9.00 ± 2.92^a^	18.18 ± 3.36^a^
2	Water + *Bti*(50)* + *food	6.67 ± 2.22^a^	12.74 ± 3.25^ab^	23.48 ± 4.10^a^
3	*Euphorbia prostrata + *water* + *food	34.06 ± 5.45^a^	42.70 ± 5.28^ab^	51.64 ± 6.25^ab^
4	*Euphorbia prostrate + Bti *10 ppm	37.22 ± 3.13^a^	69.66 ± 4.49^b^	74.85 ± 3.97^b^
5	*Euphorbia prostrate + Bti *50 ppm	39.16 ± 4.66^a^	84.85 ± 3.76^b^	88.31 ± 4.11^b^
6	*Euphorbia prostrata + Bti *100 ppm	63.50 ± 4.23^a^	90.94 ± 2.98^b^	97.54 ± 0.56^c^
7	*Euphorbia prostrata + Bti *250 ppm	79.31 ± 4.27^a^	96.54 ± 2.20^b^	100.00^c^

a, b, c, and ab: means sharing the same letter within each treatment are not statistically different.

**Table 5:** Toxicity of different concentrations of *Bti in *combination with 30% *Euphorbia prostrata* extract.

Plants	Observation (hrs later)	*N*	LC_50_ (lower value ± upper value)	Slope ± SE	*X* ^2^	*P* value
*Bti + Euphorbia prostrate*	24	20	75.53 (46.32 ± 101.12)	0.0050047 ± 0.0007578	4.30	0.01
	48	20	34.96 (22.60 ± 84.90)	0.0058996 ± 0.0012481	3.27	0.00
	72	20	12.49 (8.02 ± 70.72)	0.0146966 ± 0.0031638	0.26	0.00

^**∗**^10, 50, 100, and 250 ppm concentrations of *Bti*.

**Table 6:** Effect of different concentrations of weed plant extracts on the percent inhibition of enzymatic activity in *Drosophila melanogaster *larvae.

Plants	AChE			AcP			AkP			*α*-Carboxyl			*β*-Carboxyl		
	10%	20%	30%	10%	20%	30%	10%	20%	30%	10%	20%	30%	10%	20%	30%
*E. prostrata*	21.33 ± 2.12^a^	37.56 ± 3.03^b^	53.72 ± 1.37^c^	17.68 ± 1.08^a^	22.38 ± 1.42^b^	33.32 ± 1.15^c^	9.76 ± 0.79^a^	14.77 ± 0.79^b^	23.61 ± 1.19^c^	38.35 ± 2.19^a^	57.86 ± 2.93^b^	70.39 ± 3.11^c^	31.01 ± 2.89^a^	47.48 ± 3.01^b^	61.32 ± 1.73^c^
	(*F* = 61.21; d.f. = 2; *P* < 0.05)			(*F* = 49.91; d.f. = 2; *P* < 0.05)			(*F* = 52.06; d.f. = 2; *P* < 0.05)			(*F* = 53.11; d.f. = 2; *P* < 0.05)			(*F* = 29.82; d.f. = 2; *P* < 0.05)		

*C. murale*	18.54 ± 2.5^a^	30.13 ± 3.35^b^	60.13 ± 2.58^c^	16.31 ± 1.47^a^	21.17 ± 1.01^b^	30.19 ± 1.31^c^	9.04 ± 1.18^a^	13.10 ± 0.98^b^	25.15 ± 0.71^c^	23.97 ± 2.15^a^	32.99 ± 4.14^a^	44.71 ± 3.61^b^	25.92 ± 3.47^a^	40.11 ± 3.06^b^	50.11 ± 2.59^b^
	(*F* = 91.37; d.f. = 2; *P* < 0.05)			(*F* = 39.96; d.f. = 2; *P* < 0.05)			(*F* = 129.31; d.f. = 2; *P* < 0.05)			(*F* = 12.89; d.f. = 2; *P* < 0.05)			(*F* = 15.11; d.f. = 2; *P* < 0.05)		

*A. indica*	15.93 ± 2.88^a^	33.19 ± 3.56^b^	53.01 ± 2.45^c^	15.73 ± 1.81^a^	20.97 ± 1.53^b^	31.06 ± 1.17^c^	9.19 ± 1.11^a^	13.10 ± 0.81^a^	20.99 ± 1.57^b^	32.11 ± 2.07^a^	53.07 ± 3.11^b^	64.17 ± 1.76^c^	27.18 ± 2.35^a^	45.96 ± 3.44^b^	47.41 ± 3.13^b^
	(*F* = 93.79; d.f. = 2; *P* < 0.05)			(*F* = 31.14; d.f. = 2; *P* < 0.05)			(*F* = 25.69; d.f. = 2; *P* < 0.05)			(*F* = 79.14; d.f. = 2; *P* < 0.05)			(*F* = 22.95; d.f. = 2; *P* < 0.05)		

*F. indica*	21.15 ± 2.93^a^	28.18 ± 2.47^a^	50.56 ± 2.82^b^	18.78 ± 1.53^a^	22.43 ± 1.54^b^	30.19 ± 1.35^c^	10.17 ± 1.51^a^	12.68 ± 1.63^a^	25.55 ± 1.48^b^	27.85 ± 2.61^a^	44.72 ± 2.84^b^	46.80 ± 3.69^b^	21.44 ± 4.99^a^	34.14 ± 3.68^ab^	48.77 ± 3.91^b^
	(*F* = 46.15; d.f. = 2; *P* < 0.05)			(*F* = 43.41; d.f. = 2; *P* < 0.05)			(*F* = 32.54; d.f. = 2; *P* < 0.05)			(*F* = 13.65; d.f. = 2; *P* < 0.05)			(*F* = 11.31; d.f. = 2; *P* < 0.05)		

*P. hysterophorus*	12.17 ± 2.71^a^	23.42 ± 2.61^b^	49.59 ± 1.93^c^	18.80 ± 1.42^a^	22.31 ± 1.72^a^	27.97 ± 1.28^b^	9.52 ± 1.23^a^	15.66 ± 1.47^b^	24.41 ± 1.31^c^	23.19 ± 2.73^a^	35.81 ± 3.17^b^	46.78 ± 3.44^c^	15.85 ± 3.79^a^	35.94 ± 3.55^b^	46.88 ± 2.57^b^
	(*F* = 61.68; d.f. = 2; *P* < 0.05)			(*F* = 11.18; d.f. = 2; *P* < 0.05)			(*F* = 40.23; d.f. = 2; *P* < 0.05)			(*F* = 22.41; d.f. = 2; *P* < 0.05)			(*F* = 22.41; d.f. = 2; *P* < 0.05)		

AChE: acetylcholinesterase, AcP: acid phosphatases, AkP: alkaline phosphatases, *α*-Carboxyl: *α*-carboxylesterases, and *β*-Carboxyl: *β*-carboxylesterases; means sharing the same letter within each treatment are not statistically different.

**Table 7:** Percent inhibition of enzyme activity in *Drosophila melanogaster *larvae using different concentrations of *Bti *at 30% concentrations of *Euphorbia prostrata*.

Concentrations	A Ch E	AcP	AkP	*α*-Carboxyl	*β*-Carboxyl
	(*F* = 25.92; d.f. = 5; *P* < 0.05)	(*F* = 24.23; d.f. = 5; *P* < 0.05)	(*F* = 53.78; d.f. = 5; *P* < 0.05)	(*F* = 10.68; d.f. = 5; *P* < 0.05)	(*F* = 6.79; d.f. = 5; *P* < 0.05)
Water* + *food	3.69 ± 1.16^a^	0.59 ± 1.22^a^	0.00 ± 0.67^a^	0.00 ± 0.66^a^	0.61 ± 0.90^a^
Water + *Bti + *food	6.12 ± 0.83^a^	0.96 ± 1.11^b^	0.85 ± 0.76^a^	1.07 ± 1.12^a^	1.57 ± 1.16^a^
*Euphorbia prostrata + *water* + *food	53.72 ± 1.37^c^	33.32 ± 1.15^c^	23.61 ± 1.19^c^	70.39 ± 3.11^c^	61.32 ± 1.73^c^
*Euphorbia prostrate + Bti *10 ppm	11.57 ± 1.20^bc^	7.10 ± 1.32^ac^	7.10 ± 1.32^ac^	2.78 ± 1.07^a^	3.64 ± 1.33^a^
*Euphorbia prostrate + Bti *50 ppm	14.12 ± 1.38^cd^	8.13 ± 1.41^a^	5.59 ± 1.45^b^	2.56 ± 1.37^a^	3.03 ± 1.38^a^
*Euphorbia prostrata + Bti *100 ppm	16.67 ± 0.97^de^	10.77 ± 1.72^a^	10.70 ± 1.69^c^	7.27 ± 1.77^a^	6.67 ± 1.01^ab^
*Euphorbia prostrata + Bti *250 ppm	20.23 ± 1.38^e^	16.47 ± 2.08^d^	19.22 ± 3.14^d^	15.17 ± 3.15^b^	13.33 ± 3.14^b^

AChE: acetylcholinesterase, AcP: acid phosphatases, AkP: alkaline phosphatases, *α*-Carboxyl: *α*-carboxylesterases, and *β*-Carboxyl: *β*-carboxylesterases; means sharing the same letter within each treatment are not statistically different.

**Table 8:** Phytochemical constituents of petroleum ether extracts of selected weed plants.

Sr. number	Weed plants	Chemical constituents							
		Fl	Sa	Tn	St	CG	Al	Anth	Ter
1	*E. prostrata *	+	+	+	−	+	+	+	−
2	*C. murale*	+	+	+	+	+	+	−	−
3	*A. indica*	+	+	+	+	+	+	+	+
4	*F. indica*	+	+	−	+	−	+	+	+
5	*P. hysterophorus*	+	+	−	+	+	+	+	+

Fl: flavonoids, Sa: saponins, Tn: tannins, St: steroids, CG: cardiac glycosides, Al: alkaloids, Anth: anthraquinones, and Ter: terpenoids; +: positive; −: negative.
